# Supplementary material for: The Association between Cardiovascular Risk Factors and Lichen Sclerosus: A Systematic Review and Meta-Analysis
Source: J Clin Med. 2024 Aug 9;13(16):4668. doi: 10.3390/jcm13164668 (PMC11355417; doi:10.3390/jcm13164668)
Supplement: Supplementary file 1 [file jcm-13-04668-s001.zip › Table S3.pdf]

Table S3: Search Strategy

Database: MEDLINE

1. lichen sclerosus.mp. or exp Lichen Sclerosus et Atrophicus/
2. obesity.mp. or exp Obesity/
3. overweight.mp. or exp Overweight/
4. body mass index.mp. or exp Body Mass Index/
5. obese.mp.
6. bmi.mp.
7. or/2-6
8. metabolic syndrome.mp. or exp Metabolic Syndrome/
9. blood pressure$.mp. or exp Blood Pressure/
10. hypertension.mp or exp Hypertension/
11. hypertensive$.mp
12. systolic$.mp
13. diastolic$.mp
14. or/9-13
15. diabetes mellitus.mp. or exp Diabetes Mellitus/
16. diabetes.mp.
17. exp Diabetes Mellitus, Type 1/ or exp Diabetes Mellitus, Type 2/
18. hyperglycemia.mp. or exp Hyperglycemia/
19. hyperglycaemia.mp.
20. exp Insulin Resistance/
21. or/15-20
22. dyslipid$.mp. or exp Dyslipidemia/
23. hyperlipid$.mp. or exp Hyperlipidemia/
24. hypercholesterol$ or exp Hypercholesterolemia/
25. hypertriglyceridemia.mp. or exp Hypertriglyceridemia/
26. or/22-25
27. exp Hypothyroidism/ or hypothyroid*.mp.
28. exp Hyperthyroidism/ or hyperthyroid*.mp. or exp Graves Disease/
29. exp Autoimmune Diseases/ or autoimmune.mp. or exp Thyroiditis, Autoimmune/
30. exp Thyroid Diseases/ or thyroid*.mp.
31. exp Hashimoto Disease/ or Hashimoto*.mp. or exp Thyroiditis, Autoimmune/
32. or/27-31
33. 1 and (7 or 8 or 14 or 21 or 26 or 32)

Database: Embase

1. 'lichen sclerosus et atrophicus'/exp OR 'lichen sclerosus et atrophicus'
2. 'obesity' or 'Obesity'/exp
3. 'overweight' or 'Overweight'/exp
4. 'body mass index' or 'Body Mass Index'/exp
5. 'obese'
6. 'bmi'
7. #2 OR #3 OR #4 OR #5 OR #6
8. 'metabolic syndrome*' or 'Metabolic Syndrome'/exp
9. 'blood pressure*' or 'Blood Pressure'/exp
10. 'hypertension' or 'Hypertension'/exp
11. 'hypertensive*'
12. 'systolic*'
13. 'diastolic*'
14. #9 OR #10 OR #11 OR #12 OR #13
15. 'diabetes mellitus' or 'Diabetes Mellitus'/exp
16. 'diabetes'
17. 'Diabetes Mellitus, Type 1'/exp or 'Diabetes Mellitus, Type 2'/exp
18. 'hyperglycemia' or 'Hyperglycemia'/exp
19. 'hyperglycaemia'
20. 'Insulin Resistance'/exp
21. #15 OR #16 OR #17 OR #18 OR #19 OR #20
22. 'dyslipid*' or 'Dyslipidemia'/exp
23. 'hyperlipid*' or 'Hyperlipidemia'/exp
24. 'hypercholesterol*' or 'Hypercholesterolemia'/exp
25. 'hypertriglyceridemia' or 'Hypertriglyceridemia'/exp
26. #22 OR #23 OR #24 OR #25
27. 'thyroid disease'/exp OR 'thyroid disease'
28. 'hypothyroidism'/exp OR 'hypothyroidism'
29. 'hyperthyroidism'/exp OR 'hyperthyroidism'
30. 'thyroiditis'/exp OR 'thyroiditis'
31. 'anti tpo'
32. 'hashimoto disease'/exp OR 'hashimoto disease'
33. 'graves disease'/exp OR 'graves disease'
34. autoimmune
35. #27 OR #28 OR #29 OR #30 OR #31 OR #32 OR #33 OR #34
36. #1 AND (#7 OR #14 OR #21 OR #26 OR #35)
