# Supplementary material for: The Association between Cardiovascular Risk Factors and Lichen Sclerosus: A Systematic Review and Meta-Analysis
Source: J Clin Med. 2024 Aug 9;13(16):4668. doi: 10.3390/jcm13164668 (PMC11355417; doi:10.3390/jcm13164668)
Supplement: Supplementary file 1 [file jcm-13-04668-s001.zip › Table S4.pdf]

Table S4: Diagnostic criteria of patients with lichen sclerosus in each of the included studies

References	Diagnostic criteria
Bjekić 2011 [16]	Diagnosis was made by history and physical examination by a dermatologist.
Blaschko 2015 [28]	Diagnosis was identified through ICD-9 codes for LS (701.0, 607.81, 697.8, 697.7) in the Nationwide Inpatient Sample.
Cooper 2008 [29]	Diagnosis was based on the typical clinical features of adult-onset LS of the vulva. This included a personal history of autoimmune disorder, a family history of autoimmune disorder, and an autoantibody screen. Specific antibodies tested included thyroid peroxidase, antinuclear, antigastric parietal, anti-smooth muscle, and antimitochondrial antibodies.
Elkhoury 2023 [30]	Diagnosis was confirmed by pathology following circumcision.
Erickson 2015 [14]	Diagnosis was made by visualization of classic dermatologic characteristics, including whitish skin, tissue fusion, scarring, and chronic irritation. Confirmatory biopsies were performed in 64% of cases to rule out malignancy.
Fuchs 2017 [31]	Diagnosis was confirmed by pathology report following circumcision.
Gulin 2023 [13]	Diagnosis was identified using ICD-10 code L90.0 from the journal software used by the healthcare provider
Halonen 2024 [18]	Diagnosis was identified from the Care Register for Health Care (HILMO) using the ICD-10 code L90.0 for lichen sclerosus.
Hieta 2021 [17]	Diagnosis was identified using the ICD-10 code L90.0 from hospital patient data in Turku University Hospital. The diagnosis was clinical or confirmed with a skin biopsy.
Higgins 2012 [32]	Diagnosis was based on clinical examination and, where applicable, confirmed by histopathology.

References	Diagnostic criteria
Hofer 2014 [15]	Diagnosis was made through histologic confirmation of LS.
Hu 2020 [33]	Diagnosis was based on symptoms, physical examination, and vulvar biopsy in ambiguous cases.
Meeks 2011 [34]	Diagnosis was made through histologic confirmation of LS.
Meyrick thomas 1983 [35]	Diagnosis was made through histologic confirmation of LS.
Ranum 2022 [20]	Diagnosis was identified using specific ICD-9 or ICD-10 codes. The genital LS subgroup was defined as patients with at least genital involvement, and the extragenital LS subgroup was defined as patients with only extragenital involvement.
Yen Luu 2023 [19]	Diagnosis was made based on clinical examination and confirmed by histopathology in ambiguous cases.

Abbreviations: ICD, International Classification of Diseases; LS, lichen sclerosus; NR, Not reported.
