# Supplementary material for: The Association between Cardiovascular Risk Factors and Lichen Sclerosus: A Systematic Review and Meta-Analysis
Source: J Clin Med. 2024 Aug 9;13(16):4668. doi: 10.3390/jcm13164668 (PMC11355417; doi:10.3390/jcm13164668)
Supplement: Supplementary file 1 [file jcm-13-04668-s001.zip › Table S5.pdf]

Table S5: Severity of lichen sclerosus and criteria for diagnosis of diabetes mellitus, hypertension, dyslipidemia, obesity, and metabolic syndrome among included studies

References	LS severity	Treatments of LS	Type of DM	Criteria for DM type 1	Criteria for DM type 2	Criteria for HT	Criteria for dyslipidemia	Criteria for obesity	Criteria for metabolic syndrome
Bjekić 2011 [16]	NR	NR	NR	NR	NR	NA	NR	NA	NA
Blaschko 2015 [28]	NR	NR	NA	NA	NA	NR	NA	NR	NA
Cooper 2008 [29]	NR	NR	1	NR	NR	NA	NA	NA	NA
Elkhoury 2023 [30]	NR	NR	NR	NR	NR	NR	NA	NA	NA
Erickson 2015 [14]	NR	NR	NR	NR	NR	NR	NR	NA	NA
Fuchs 2017 [31]	NR	NR	NA	NA	NA	NA	NA	Defined using the Centers for Disease Control and Prevention BMI-for-age growth chart, characterized as BMI ≥95th percentile for age, and overweight was characterized as BMI 85th to 94th percentile for age	NA
Gulin 2023 [13]	NR	NR	1	NR	NR	NA	NA	NA	NA
Halonen 2024 [18]	NR	NR	1,2	NR	NR	NR	NR	NR	NR
Hieta 2021 [17]	NR	NR	2	NR	NR	NR	NR	NA	NA
Higgins 2012 [32]	NR	NR	1, 2	NR	NR	NA	NA	NA	NA
Hofer 2014 [15]	NR	NR	1, 2	NR	NR	NR	NR	NA	NA
Hu 2020 [33]	NR	NR	NA	NA	NA	NR	NA	NA	NA
Meeks 2011 [34]	NR	NR	NR	NR	NR	NA	NA	NA	NA
Meyrick thomas 1983 [35]	NR	NR	2	NR	NR	NA	NA	NA	NA
Ranum 2022 [20]	NR	NR	2	NR	NR	NR	NA	NA	Defined according to the International Diabetes Federation consensus: BMI > 30 kg/m² plus any two of the following criteria: triglycerides > 150 mg/dL, HDL cholesterol < 40 mg/dL in men or < 50 mg/dL in women, diagnosed HT, or DM type 2
Yen Luu 2023 [19]	Grade 2 (50.1%), Grade 3 (47.7%)	NR	NR	NR	NR	NA	NA	NA	NA

Abbreviations: BMI, body mass index**;** DM, diabetes mellitus; HDL, high-density lipoproteins; HT, hypertension; LS, lichen sclerosus; NA, not applicable; NR, not reported.

Studies not included in the meta-analysis for each outcome were marked as NA.
