# Supplementary material for: The Association between Cardiovascular Risk Factors and Lichen Sclerosus: A Systematic Review and Meta-Analysis
Source: J Clin Med. 2024 Aug 9;13(16):4668. doi: 10.3390/jcm13164668 (PMC11355417; doi:10.3390/jcm13164668)
Supplement: Supplementary file 1 [file jcm-13-04668-s001.zip › Table S6.pdf]

Table S6: The risk of bias using The Quality In Prognosis Studies (QUIPS) tool for included articles

References	Study participant	Study attrition	Prognostic factor measurements	Outcome measurement	Confounding	Statistical analysis and reporting	Summary
Bjekić 2011 [16]	Low	Low	Low	Low	Moderate	Low	Moderate
Blaschko 2015 [28]	Low	Moderate	Low	Low	Low	Low	Moderate
Cooper 2008 [29]	Low	Low	Low	Low	Low	Low	Low
Elkhoury 2023 [30]	Low	Moderate	Low	Low	Low	Low	Moderate
Erickson 2015 [14]	Low	Low	Low	Low	Low	Low	Low
Fuchs 2017 [31]	Low	Low	Low	Low	Low	Low	Low
Gulin 2023 [13]	Low	Moderate	Low	Low	Low	Low	Moderate
Halonen 2024 [18]	Low	Low	Low	Low	Moderate	Low	Moderate
Hieta 2021 [17]	Low	Low	Low	Low	Moderate	Low	Moderate
Higgins 2012 [32]	Low	Low	Low	Low	High	Low	Moderate
Hofer 2014 [15]	Low	Low	Low	Low	Low	Low	Low
Hu 2020 [33]	Low	Moderate	Low	Low	Low	Low	Moderate
Meeks 2011 [34]	Low	Low	Low	Low	High	Low	Moderate
Meyrick thomas 1983 [35]	Low	Moderate	Low	Low	Low	Low	Moderate
Ranum 2022 [20]	Low	Moderate	Low	Low	Moderate	Low	Moderate
Yen Luu 2023 [19]	Low	Low	Low	Low	Moderate	Low	Moderate
