# Supplementary material for: The Association between Cardiovascular Risk Factors and Lichen Sclerosus: A Systematic Review and Meta-Analysis
Source: J Clin Med. 2024 Aug 9;13(16):4668. doi: 10.3390/jcm13164668 (PMC11355417; doi:10.3390/jcm13164668)
Supplement: Supplementary file 1 [file jcm-13-04668-s001.zip › Table S7.pdf]

**Table S7:** GRADE (Grading of Recommended Assessment, Development, and Evaluation) assessment of the eligible studies

| Certainty assessment | | | | | | | Certainty | Importance |
|---|---|---|---|---|---|---|---|---|
| **Outcome** | **Number of eligible studies** | **Risk of bias** | **Inconsistency** | **Indirectness** | **Imprecision** | **Publication bias** | | |
| Diabetes mellitus | 13 | not serious | not serious | not serious | not serious | suspected | ⊕⊕◯◯ Low | The risk of diabetes mellitus is probably higher in patients with lichen sclerosus. |
| Hypertension | 9 | not serious | not serious | not serious | not serious | not applicable | ⊕⊕◯◯ Low | It is possible that the risk of hypertension is increased in patients with lichen sclerosus. |
| Dyslipidemia | 5 | not serious | not serious | not serious | serious | not applicable | ⊕◯◯◯ Very low | It is unlikely that the risk of dyslipidemia is significant in patients with lichen sclerosus. |
| Obesity | 3 | not serious | not serious | not serious | serious | not applicable | ⊕◯◯◯ Very low | It is unlikely that the risk of obesity is significant in patients with lichen sclerosus. |
| Metabolic syndrome | 2 | not serious | not serious | not serious | serious | not applicable | ⊕◯◯◯ Very low | It is unlikely that the risk of metabolic syndrome is significant in patients with lichen sclerosus. |
