# Supplementary figures and images for: The Association between Cardiovascular Risk Factors and Lichen Sclerosus: A Systematic Review and Meta-Analysis
Source: J Clin Med. 2024 Aug 9;13(16):4668. doi: 10.3390/jcm13164668 (PMC11355417; doi:10.3390/jcm13164668)

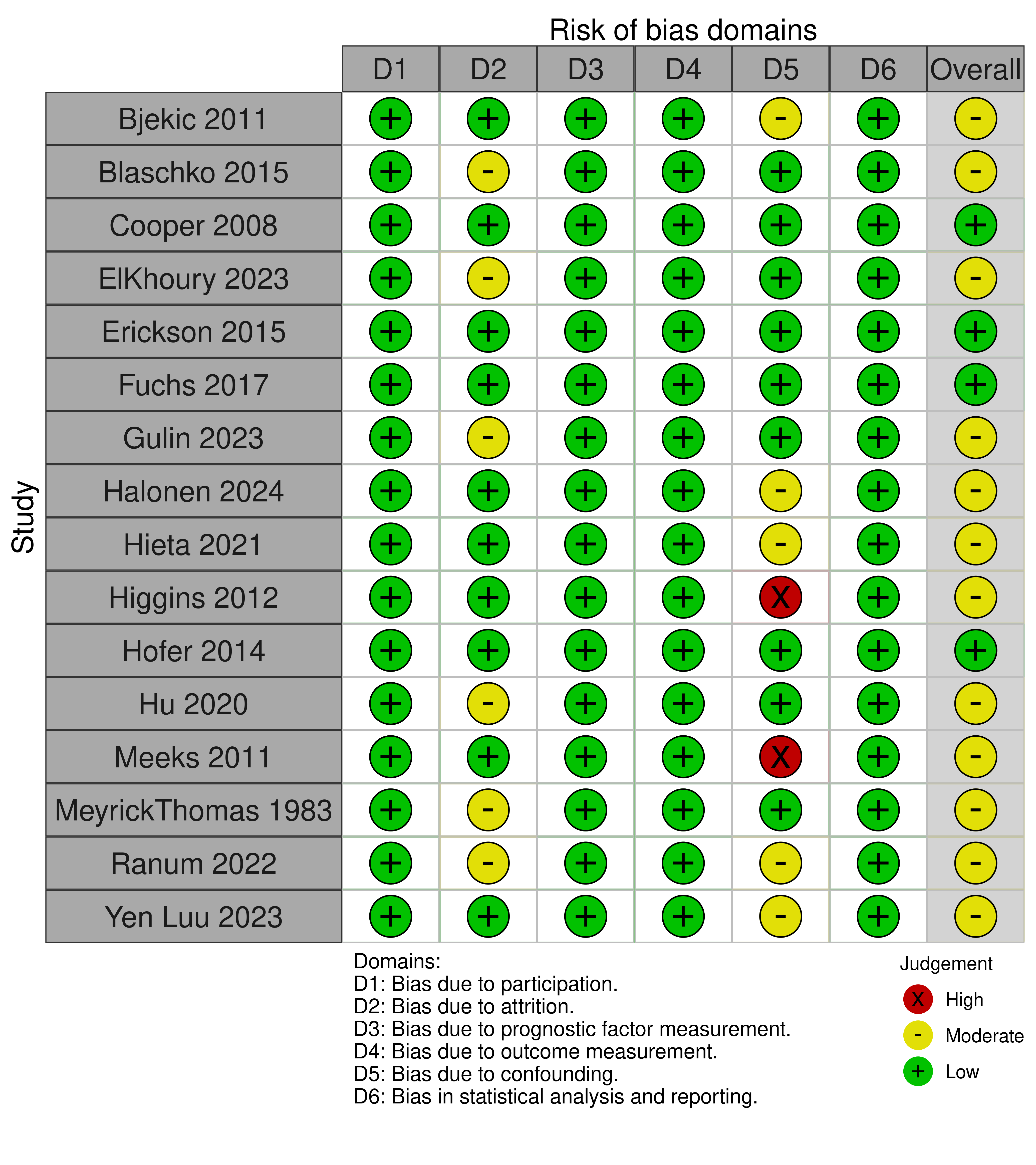

Supplement: Supplementary file 1 [file jcm-13-04668-s001.zip › Fig. S1.png]

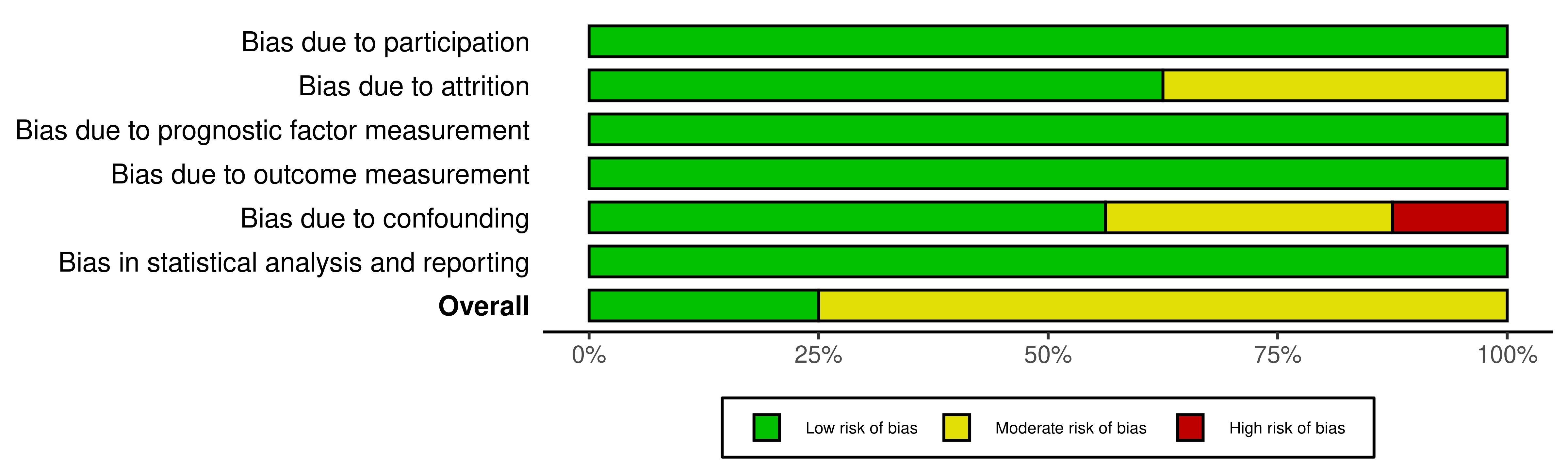

Supplement: Supplementary file 1 [file jcm-13-04668-s001.zip › Fig. S2.png]

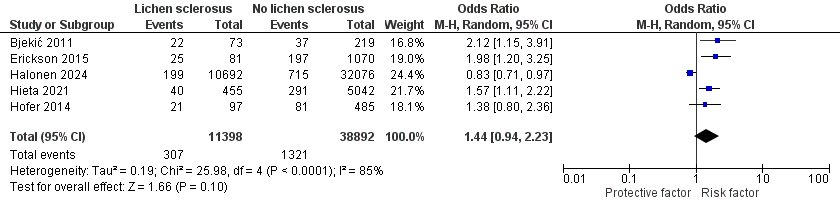

Supplement: Supplementary file 1 [file jcm-13-04668-s001.zip › Fig. S3.png]

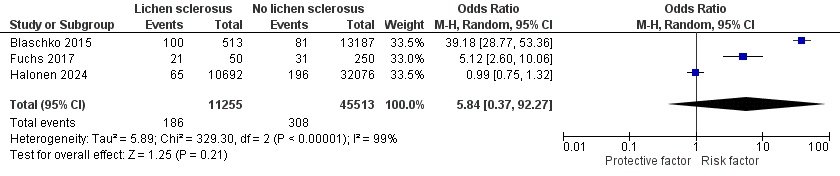

Supplement: Supplementary file 1 [file jcm-13-04668-s001.zip › Fig. S4.png]

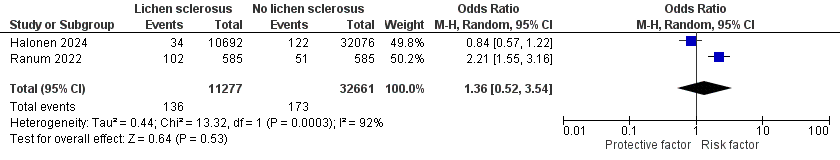

Supplement: Supplementary file 1 [file jcm-13-04668-s001.zip › Fig. S5.png]

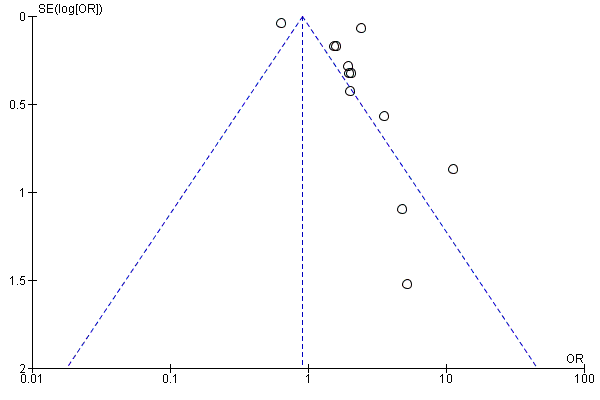

Supplement: Supplementary file 1 [file jcm-13-04668-s001.zip › Fig. S6.png]
